# Self-assembled epitope-based nanoparticles targeting the SARS-CoV-2 spike protein enhanced the immune response and induced potential broad neutralizing activity

**DOI:** 10.3389/fcimb.2025.1560330

**Published:** 2025-04-09

**Authors:** Yue Liu, Chenxi Li, Zirui Wu, Yu Zhao, Tieyan Yin, Xiaopan Liu, Jiaru Hui, Qingyu Wang, Yi Pan, Yaming Shan, Xinglong Qu

**Affiliations:** ^1^ Department of Echocardiography, The First Hospital of Jilin University, Changchun, China; ^2^ Institute of Virology and AIDS Research, The First Hospital of Jilin University, Changchun, China; ^3^ National Engineering Laboratory for AIDS Vaccine, School of Life Sciences, Jilin University, Changchun, China; ^4^ Key Laboratory for Molecular Enzymology and Engineering, The Ministry of Education, School of Life Sciences, Jilin University, Changchun, China; ^5^ Department of Respiratory, The First Hospital of Jilin University, Changchun, China

**Keywords:** SARS-CoV-2, ferritin, self-assembled nanoparticles, key epitopes, immunogenicity study

## Abstract

**Introduction:**

The ongoing COVID-19 has caused a global pandemic, resulting in millions of infections and deaths. While current vaccines target the SARS-CoV-2 spike (S) protein, its high mutation rate significantly compromises vaccine efficacy. We aimed to evaluate the potential of epitope-based nanoparticles (NPs) to induce broad cross-protection and durable immune responses against SARS-CoV-2.

**Methods:**

Four conserved epitopes derived from the receptor-binding domain (RBD) and S2 subunit of the spike protein were integrated into *Helicobacter pylori* ferritin to create epitope-based NPs named S18-F, RBM-F, UH-F, and HR2-F. The immunogenicity of the epitope-based NPs was evaluated through animal experiments to measure epitope-specific antibody titers and assess neutralizing activity against SARS-CoV-2 pseudovirus. To characterize cellular immune responses, splenic lymphocyte proliferation following epitope stimulation was measured, and cytokine secretion profiles including IFN-γ, IL-2, IL-4, and IL-10 were analyzed to determine Th1/Th2 immune polarization. Antibody-dependent cellular cytotoxicity (ADCC) assays were performed to evaluate NP-enhanced recognition and elimination of infected target cells.

**Results:**

These NPs induced high titers of epitope-specific antibodies lasting three months post-immunization. Sera from the RBM-F, UH-F, and HR2-F groups exhibited neutralizing activity against the SARS-CoV-2 pseudovirus WH-1 *in vitro*. Splenic lymphocytes from the S18-F, RBM-F, and UH-F groups showed significantly increased proliferation. Lymphocytes from the RBM-F group demonstrated increased secretion of IFN-γ, IL-2, IL-4, and IL-10 cytokines, indicating a balanced Th1 and Th2 immune response. Immune sera from the S18-F and mixed-immunized groups exhibited antibody-dependent cellular cytotoxicity.

**Discussion:**

The results indicate that these NPs induce robust humoral and cellular immune responses, potentially offering a promising strategy for effective vaccine development against SARS-CoV-2.

## Graphical Abstract

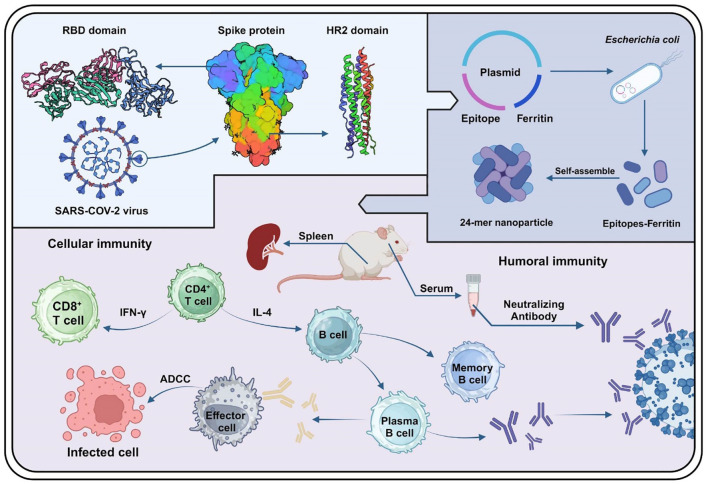

## 1 Introduction

At the end of 2019, COVID-19 emerged as a global pandemic (Rothan and Byrareddy, 2020; Zhu et al., 2020). The International Committee on Taxonomy of Viruses identified the causative pathogen as SARS-CoV-2 (The species Severe acute respiratory syndrome-related coronavirus: classifying 2019-nCoV and naming it SARS-CoV-2, 2020). Phylogenetic analysis revealed that SARS-CoV-2 shares 79% sequence similarity with SARS-CoV and over 95% homology with the bat coronavirus RaTG13 (Lu et al., 2020). According to statistics of World Health Organization, SARS-CoV-2 has resulted in approximately 700 million infections and over six million deaths.

Existing vaccines have effectively prevented SARS-CoV-2 infection, playing a crucial role in controlling viral transmission and alleviating symptoms (Tregoning et al., 2021). These vaccines target the spike (S) protein, eliciting protective immune responses (Li et al., 2022). However, the high mutation rate of the S protein has led to reduced alignment with emerging variants, which exposed the limitations of traditional drug discovery methods (Zhang et al., 2022). Researchers increasingly rely on bioinformatics analyses for the development of vaccines and antibodies. Bioinformatics has proven invaluable in predicting numerous conserved epitopes (Khan et al., 2023). Recent findings emphasize the critical roles of linear epitopes in virus vulnerability and conservation, as well as their importance as components of conformational epitopes necessary for inducing virus neutralization. For example, a linear epitope of Human Immunodeficiency Virus (HIV) demonstrated broad-spectrum protective effects and could be pivotal in developing universal vaccines (Kong et al., 2019). Grifoni et al. identified potential B and T cell epitopes for SARS-CoV-2, which hold promise as targets for immune recognition against the virus (Grifoni et al., 2020).

The S protein is integral to viral host cell infection and comprises two subunits, S1 and S2. The receptor-binding domain (RBD) of S1 contains a core structure and a receptor-binding motif (RBM) that interacts with Angiotensin-Converting Enzyme 2 (ACE2). Subunit S2 consists of the heptad repeat (HR1), heptad repeat 2 (HR2), and the central helix (CH), which mediate virus-cell membrane fusion (Shang et al., 2020). Consequently, RBD and S2 are primary targets for designing vaccine candidates based on the S protein (Huang et al., 2020). Epitope-based vaccine strategies have demonstrated stronger immune responses and improved safety compared with those based on the full-length S protein (Soria-Guerra et al., 2015). Most neutralizing antibodies against SARS-CoV-2 primarily target the S1 subunit, particularly the RBD, to block virus attachment to host cells (Barnes et al., 2020). The RBD remains a key focus for COVID-19 vaccines aimed at eliciting neutralizing antibodies (Dickey et al., 2022). In contrast, the S2 subunit exhibits greater sequence conservation across various SARS-CoV-2 variants of concern compared to S1 (Olukitibi et al., 2023). Given that the structural transition of the S protein from prefusion to post-fusion states is critical for virus-host membrane fusion, antibodies directed against the S2 stem helix region are expected to impede SARS-CoV-2 infection (Wang et al., 2021). Recent studies have highlighted that antibodies targeting specific epitopes within the S2 subunit of the SARS-CoV-2 S protein frequently demonstrate broad neutralizing capabilities (Pinto et al., 2021; Hurlburt et al., 2022; Zhou et al., 2022).

The low immunogenicity of short peptides presents a significant challenge for epitope-based vaccine development. Nanoparticles (NPs) offer a promising solution by serving as effective delivery vehicles and immune enhancers (Walls et al., 2020; Wang et al., 2020). Among these, ferritin NPs stand out due to their excellent safety and biocompatibility (Bou-Abdallah, 2010; Truffi et al., 2016; Arosio et al., 2017; Li et al., 2023), which enhance immunogenicity by improving antigen presentation efficiency and stability. Utilizing ferritin to display trimeric conformations of the HIV gp120 protein notably enhances the immunogenicity and protective efficacy of HIV vaccines (He et al., 2016). Researchers are increasingly leveraging ferritin as a carrier based on experimental findings that consistently demonstrate enhanced immune responses. Ferritin has been successfully employed to carry various antigens, including the A helix and CD helix from the influenza virus hemagglutinin stem (Qiao et al., 2022), influenza virus hemagglutinin trimer (Kanekiyo et al., 2013), as well as full-length S protein and RBD sequences of SARS-CoV-2 (Powell et al., 2021). Studies indicate that NPs such as ferritin (24-mer), mi3 (60-mer), and I53–50 (120-mer) induce more robust immune responses compared to RBD monomers (Kang et al., 2021). Specifically, ferritin-displayed antigens from influenza A virus have demonstrated robust and efficient immune responses (Kanekiyo et al., 2013).

In this study, we developed and characterized NPs based on conserved linear epitopes from the SARS-CoV-2 S protein RBD and S2 domain. Using IEDB and Bepipred Linear Epitope Prediction 2.0, we identified four conserved epitopes (S18, RBM, UH, and HR2). These epitopes were integrated with *Helicobacter pylori* ferritin to create epitope-based NPs named S18-F, RBM-F, UH-F, and HR2-F. The immunogenicity of the epitope-based NPs was assessed through animal experiments to measure epitope-specific antibody titers and evaluate neutralizing antibody levels against the SARS-CoV-2 pseudovirus. Additionally, splenic lymphocyte proliferation in response to epitope stimulation was analyzed to assess the cellular immune response. The secretion levels of Th1/Th2-associated cytokines were assessed to determine whether the epitopes could elicit a balanced immune response. Furthermore, antibody-dependent cell-mediated cytotoxicity (ADCC) activities was measured to evaluate the NPs’ ability to stimulate splenic cells in recognizing and eliminating infected cells. We hope that epitope-based NPs induce robust humoral and cellular immune responses, potentially providing enduring protection. Utilizing epitope-based self-assembled ferritin could thus present an effective strategy for vaccine development.

## 2 Materials and methods

### 2.1 Animals and cells

Specific pathogen-free 6–8-week-old female BALB/c mice were procured from Liaoning Changsheng Biotechnology Co., Ltd. (Liaoning, China).

Human embryonic kidney 293T cells were obtained from the American Type Culture Collection (Manassas, VA, USA), and Huh7 cells were obtained from the Japanese Cancer Research Resources Bank (Tokyo, Japan). Briefly, the cells were propagated in Dulbecco’s modified Eagle’s medium (Gibco, Grand Island, NY, USA) supplemented with 10% heat-inactivated fetal bovine serum (Gibco, Grand Island, NY, USA) and 1% penicillin–streptomycin (Sinopharm Chemical Reagent, Beijing, China).

### 2.2 Conservation analysis of the RBD and S2 protein

Full-length amino acid sequences of different SARS-CoV-2 surface glycoprotein (n=37) from 2019 to 2024 were downloaded from the National Center for Biotechnology Information (NCBI) in the FASTA format. The sequences were aligned using MEGA 11. The conservation of RBD and S2 subunit was analyzed using two approaches. The aligned sequences were loaded into the MEME Suite (https://meme-suite.org/meme/) to assess amino acid frequency at each site. Alternatively, the aligned sequences were imported into Weblogo (https://weblogo.threeplusone.com/) and a logo created to indicate amino acid conservation based on the height of each individual amino acid. Four linear epitopes listed in **Table 1** were identified based on these analyses, named S18, RBM, UH, and HR2. The peptides, S18, RBM, UH and HR2 were synthesized (GL Biochem, Shanghai, China) with > 90% purity.

**Table 1:** Conservation sequences from RBD and S2 subunit.

Name	Amino Acid Sequence
RBM	SNNLDSKVGGNYNYLYRLFRKSNLKPFERDISTEIYQAGSTPCNGVEGFNCYFPLQSYGFQPTNGVGYQPY
HR2	PDVDLGDISGINASVVNIQKEIDRLNEVAKNLNESLIDLQEL
UH	VNNTVYDPLQPELDSFKEELDKYFKNHTSPDVDLGDISGI
S18	GKIADYNYKLPDDFTGCVIAWNSNNL

### 2.3 Immunogen design and gene synthesis

The genes encoding the RBM, HR2, UH, and S18 epitope sequences were cloned into pET-20b by GenScript Biotech (Nanjing, China). *Helicobacter pylori* ferritin (GenBank: NP_223316) was inserted into the plasmids at the C-terminus of the epitopes, followed by a 6 × His tag at the C-terminus of ferritin for affinity purification through the NdeI and XhoI restriction sites.

### 2.4 Protein expression and purification

The recombinant plasmids were transformed into *E. coli* BL21 (DE3) pLysS competent cells. When the *E. coli* culture entered the logarithmic growth phase, 0.1 mM isopropyl β-D-1-thiogalactopyranoside was added to induce protein expression, and the culture was maintained for another 12 hours at 16°C. The harvested cells were re-suspended in Tris-buffered saline buffer (50 mM Tris, 150 mM NaCl, pH 8.0) and disrupted by sonication at 4°C. The expressed proteins were then purified from the culture supernatant using affinity chromatography on a His-trap column (GE Healthcare, USA). The purified proteins were detected using the bicinchoninic acid method.

### 2.5 NP characterization

Sodium dodecyl sulfate-polyacrylamide gel electrophoresis (SDS-PAGE) was performed to identify the protein monomers. The samples were mixed with a four-fold loading buffer, heated for 10 min at 98°C, and separated on 12% polyacrylamide gel. A total of 5 μg of each protein was loaded onto the gel. The proteins were stained with Coomassie Brilliant Blue (Sigma, St. Louis, Missouri, USA) and decolorized in double-distilled water with 45% methanol and 10% glacial acetic acid until a clear background appeared. They were visualized and recorded using a Tanon-5200 Chemiluminescent Imaging System (Tanon Science and Technology, Shanghai, China).

The morphology of NPs was evaluated using transmission electron microscopy (TEM; H-7650, Hitachi, Japan) at an accelerating voltage of 100 kV. The diameter distribution of the NPs was determined by dynamic light scattering (DLS) at 25°C using a Zetasizer NANO ZS90 instrument (Malvern, Worcestershire, UK).

### 2.6 Animal immunization

The BALB/c mouse immunity experiment was approved from the Experimental Animal Ethics Committee of Jilin University (approval number: YN2021094). To assess the immunogenicity of the NPs, BALB/c mice were randomly assigned to 7 groups (n = 6 per group). The NPs were combined with an aluminum adjuvant (InvivoGen, San Diego, CA, USA) at a ratio of 3:1 for immunization. Mice received subcutaneous immunizations with 20 μg of ferritin, S18-F, RBM-F, UH-F, HR2-F, or a mixture of NPs. The mixed-immunized group received 5 μg of each NP to ensure consistent immunization doses across all groups. Phosphate-buffered saline (PBS) was used as a negative control. Following the initial immunization, blood was collected every 14 days, and the serum was stored at −20°C.

### 2.7 Enzyme-linked immunosorbent assay

S18, RBM, UH, and HR2 peptides (100 ng/well) were used to detect specific antibody titers. After blocking with 200 µL 5% bovine serum albumin (BSA; GENVIEW, Beijing, China) at 37°C for 2 h, serially diluted serum samples were added in duplicate and incubated at 37°C for 1 h. The plates were then incubated with horseradish peroxidase-conjugated goat anti-mouse IgG (Beijing Dingguo, China) for 1 h. Subsequently, 3,3,5,5-tetramethyl-benzidine (Tiangen Biotech, Beijing, China) was added to develop the color and the reaction was stopped using 2 M H_2_SO_4_. The absorbance at 450 nm was measured using an iMarK™ microplate reader (BioRad, Hercules, California, USA).

For the antibody isotyping assay, the ELISA plates were coated with 100 ng of S18, RBM, UH, or HR2 peptides per well. The plates were blocked with PBS containing 3% BSA, followed by incubation with serum samples (1:1000 dilution). After incubation with anti-mouse IgG1, IgG2a, IgG2b, IgG3 or IgM antibodies (1:5000 dilution; Sigma, St. Louis, Missouri, USA), alkaline phosphatase-conjugated anti-sheep IgG (Beijing Dingguo, China) was added. Subsequently, para-nitrophenyl phosphate was added as a chromogenic substrate and the reaction was terminated with 3 M NaOH. The absorbance at 405 nm was recorded immediately.

### 2.8 Splenocyte isolation and T cell activation assay

Two weeks after the final immunization, spleens were collected from the immunized mice, and splenic lymphocytes were isolated using a Mouse Spleen Lymphocyte Isolation Kit (Solarbio, Beijing, China).

The CD3^+^CD4^+^ and CD3^+^CD8^+^ T cells in the spleen were counted using flow cytometry. Briefly, splenic lymphocytes were blocked using the TruStain FcXTM PLUS (anti-mouse CD16/32) antibody (BioLegend, San Diego, California, USA). Splenic lymphocytes were incubated with fluorescein isothiocyanate-conjugated anti-mouse CD3 antibody, PE-conjugated anti-mouse CD4 antibody, or allophycocyanin-conjugated anti-mouse CD8 antibody (BioLegend, San Diego, California, USA). After washing with cell staining buffer, 5 µL of 7-aminoactinomycin D (7-AAD; BioLegend, San Diego, California, USA) was added and the number of CD3^+^CD4^+^ and CD3^+^CD8^+^ lymphocytes was determined by CytoFLEX cytometry (Beckman Coulter, Brea, California, USA).

### 2.9 Splenic lymphocyte proliferation assay

Splenocytes isolated from immunized mice were added to a 96-well culture plate (3×10^5^ cells/well). Then cells were stimulated with S18, RBM, UH, or HR2 peptides (10 µg/mL) for 72 h at 37°C. Cell proliferation was measured using the 3-(4,5-dimethylthiazol-2-yl)-2,5-diphenyltetrazolium bromide (MTT; Sigma, St. Louis, Missouri, USA) assay. Briefly, after treatment, 20 µL of MTT (5 mg/mL) was added and incubated for another 4 h in the dark. Then, 100 µL of dimethyl sulfoxide was added after removing the supernatant carefully, and the absorbance was measured at 490 nm. The stimulation index (SI) was defined as the ratio of the optical density of stimulated wells at 490 nm to that of unstimulated wells.

### 2.10 Antibody-dependent cellular cytotoxicity assay

The S protein in pCDNA3.1(+) (GENEWIZ, Beijing, China) was transfected into HKE 293T cells using polyethylenimine (PEI; Sigma, St. Louis, Missouri, USA) at a ratio of 1:4 (mg DNA/mg PEI). The transfected cells were used as target cells and incubated with 1 μM carboxyfluorescein diacetate succinimidyl ester (CFSE; Invitrogen, USA). CFSE-labeled target cells were added to 96-well plates and incubated with a 1:1000 dilution of serum samples. After incubation, splenic lymphocytes from naïve mice were used as effector cells and added to the appropriate wells. Finally, cells were stained with 7-AAD. Flow cytometry was performed using a CytoFLEX cytometer. Percent ADCC activity was calculated as follows: ADCC activity % = [(experimental lysis % – spontaneous lysis %)/(positive control lysis % – spontaneous lysis %)] ×100.

### 2.11 Amplification determined by enzyme-linked immunosorbent spot assay

The number of IFN-*γ*-secreting lymphocytes was quantified using Enzyme-Linked Immunospot Assay (ELISPOT), which assessed cytokine secretion by splenic lymphocytes isolated from the immunized mice. Splenic lymphocytes from immunized mice were added to pre-coated ImmunoSpot plates, along with S18, RBM, UH, HR2 peptides, or their mixture. Negative controls received serum-free medium. After incubation, cells were removed, and biotin-conjugated anti-IL-4 or anti-IFN-γ antibodies were added. Plates were further incubated with diluted streptavidin-horseradish peroxidase, followed by a substrate solution of 3-amino-9-ethylcarbazole to develop spots. Spot counting was performed using an ImmunoSpot analyzer (Cellular Technology Ltd., Cleveland, OH, USA).

### 2.12 Serum cytokine detection

The concentration of cytokines INF-γ, IL-2, IL-4, and IL-10 in serum was measured using mouse Th1/Th2 Uncoated ELISA Kits (Invitrogen, USA). Splenic lymphocyte suspension (500 μL) was added to 24-well plates, and the cells were stimulated with peptide S18, RBM, UH, HR2 peptides, or their mixture (10 μg/mL) at 37°C for 72 h. The culture medium was centrifuged and precipitated, the corresponding antibody was added to the supernatant, and the OD450nm value was determined using an enzyme-labeled apparatus (ELX800, Bio-TEK, Windsor, Vermont, USA).

### 2.13 Quantification of neutralizing antibodies against SARS-CoV-2 pseudotyped virus

At week 8, neutralizing activities of sera from all animals were assessed using a pseudotyped virus-based neutralization assay, as this time point demonstrated the highest titers of epitope-specific antibodies. The assay protocol was previously described in Nature Protocols (Nie et al., 2020) and conducted by Feifan Biotechnology (Jiangsu) Co., Ltd. Serum samples were serially diluted two-fold and mixed with the pseudotyped virus, followed by incubation at 37°C for 1 hour. The serum-virus mixture was then added to Huh7 cells in 96-well culture plates and incubated at 37°C for an additional 28 hours. After the incubation period, 150 μL of supernatant was aspirated from each well, and 100 μL of luciferase detection reagent was added. The reaction was allowed to proceed for 2 minutes at room temperature. Following the reaction, protein levels were detected using a multimode microplate reader (PE Ensight; Bio-TEK, Windsor, Vermont, USA). The cell control (CC) with only cells and the virus control (VC) with virus and cells are set up in each plate.

Neutralization inhibition rate = [1 - (mean luminescence intensity of sample group - mean luminescence intensity of blank control CC group)/(mean luminescence intensity of negative VC group - mean luminescence intensity of blank control CC group)] × 100%.

### 2.14 Safety evaluation of NPs *in vivo*

The immunized mice were euthanized two weeks after immunization. The main mouse tissues (heart, liver, spleen, lungs, and kidneys) were fixed in 4% paraformaldehyde. Fixed samples were embedded in paraffin, sliced, and stained with H&E.

### 2.15 Statistical analysis

Data are presented as mean ± standard deviation (SD). Groups were compared using a one-way analysis of variance (ANOVA) with Tukey’s *post hoc* test. The statistical significance compared to the ferritin group was denoted as follows: (ns (not significant), *P < 0.05, **P < 0.01, and ***P < 0.001.).

## 3 Results

### 3.1 Conservation analysis and plasmid design

**Figure 1:**
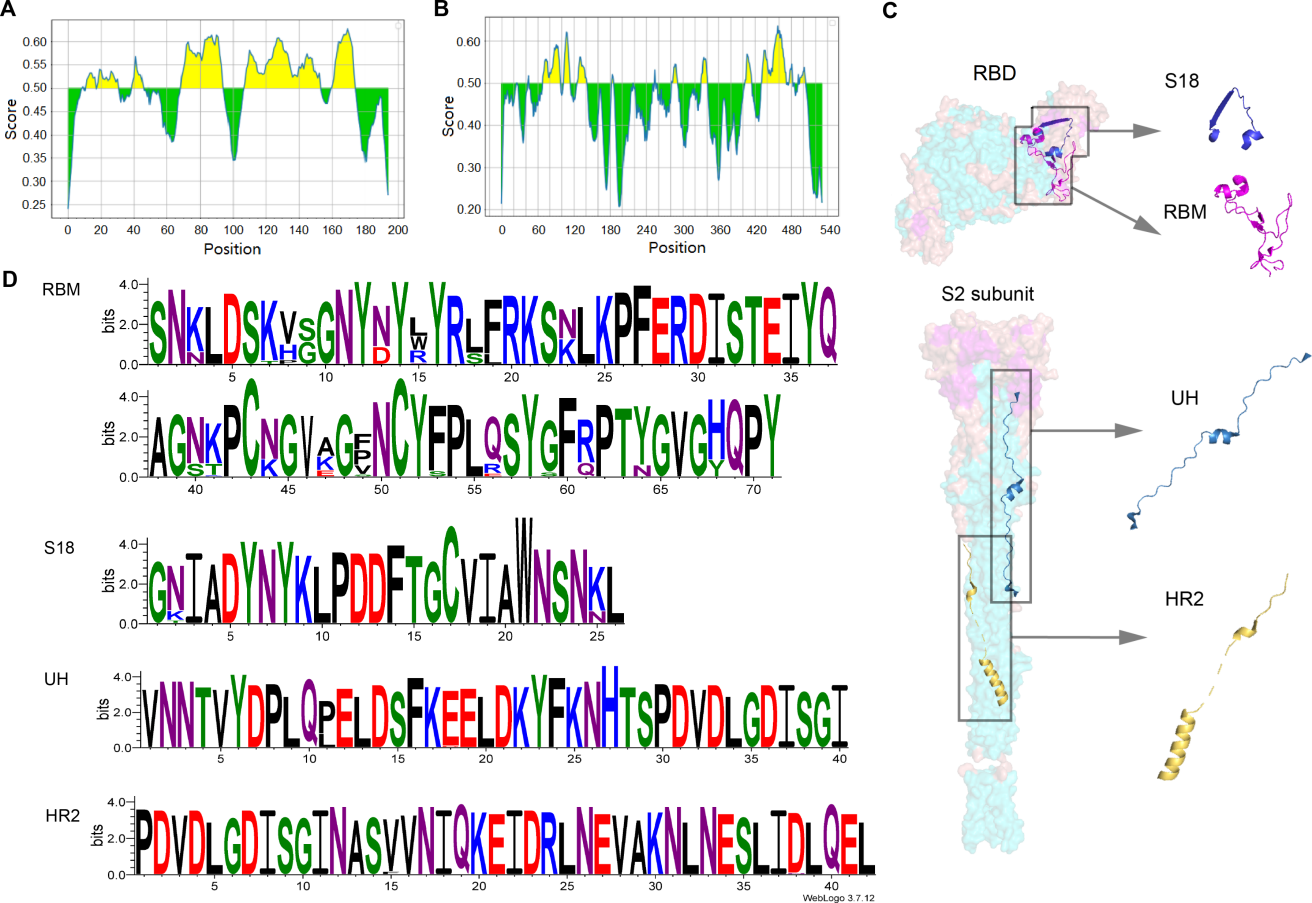
Analysis of sequence conservation and prediction of linear B-cell epitopes. **(A)** RBD and **(B)** S2 subunit linear B-cell epitope prediction using Bepipred Linear Epitope Prediction 2.0 method. **(C)** Structure RBD (ACCESSION: 7C8J_B) and S2 (ACCESSION: 7E9T_C) subunit. **(D)** Sequence logos of the multiple sequence alignments of RBM, HR2, UH and S18.

Epitope prediction prioritizes linear B-cell epitopes that are immunogenic, accessible, and preferably located within conserved regions of the protein. The conservation of RBD (PDB: 7C8J_2) and S2 (PDB: 7E9T_1) were evaluated separately from SARS-CoV-2 using IEDB and Bepipred Linear Epitope Prediction to identify potential B-cell epitopes. The B-cell epitopes in the RBD and S2 regions are depicted in **Figure 1**. Considering the surface accessibility of RBD_70-95_, RBD_84-109_ was identified as a target epitope due to its higher surface accessibility, suggesting it is more likely to act as an exposed epitope within the RBD structure. The RBM, located between RBD_106-176_, contains the epitopes RBD_108-153_ and RBD_161-174_. Given that longer segments often induce stronger immune responses, the entire length of RBM was selected as the target epitope. The allosteric folding of HR2 from S2_471-512_ is crucial during virus-target cell membrane fusion. As HR2 contains epitopes S2_478-485_ and S2_489-502_, HR2 was also chosen as a target epitope. Following comprehensive screening of various factors, four potential epitopes—RBD_84-109_, RBM, S2_431-470_, and HR2—were identified through immunoinformatics and labeled as S18, RBM, UH, and HR2. These epitopes are detailed in **Table 1** and **Supplementary Table 1**. The conservation analysis of SARS-CoV-2 variants of concern (VOCs) and variants of interest (VOIs) provided by the WHO from 2020 to 2024 was conducted using MEGA 11. The results are presented in **Supplementary Figures 1–5**.

**Figure 2:**
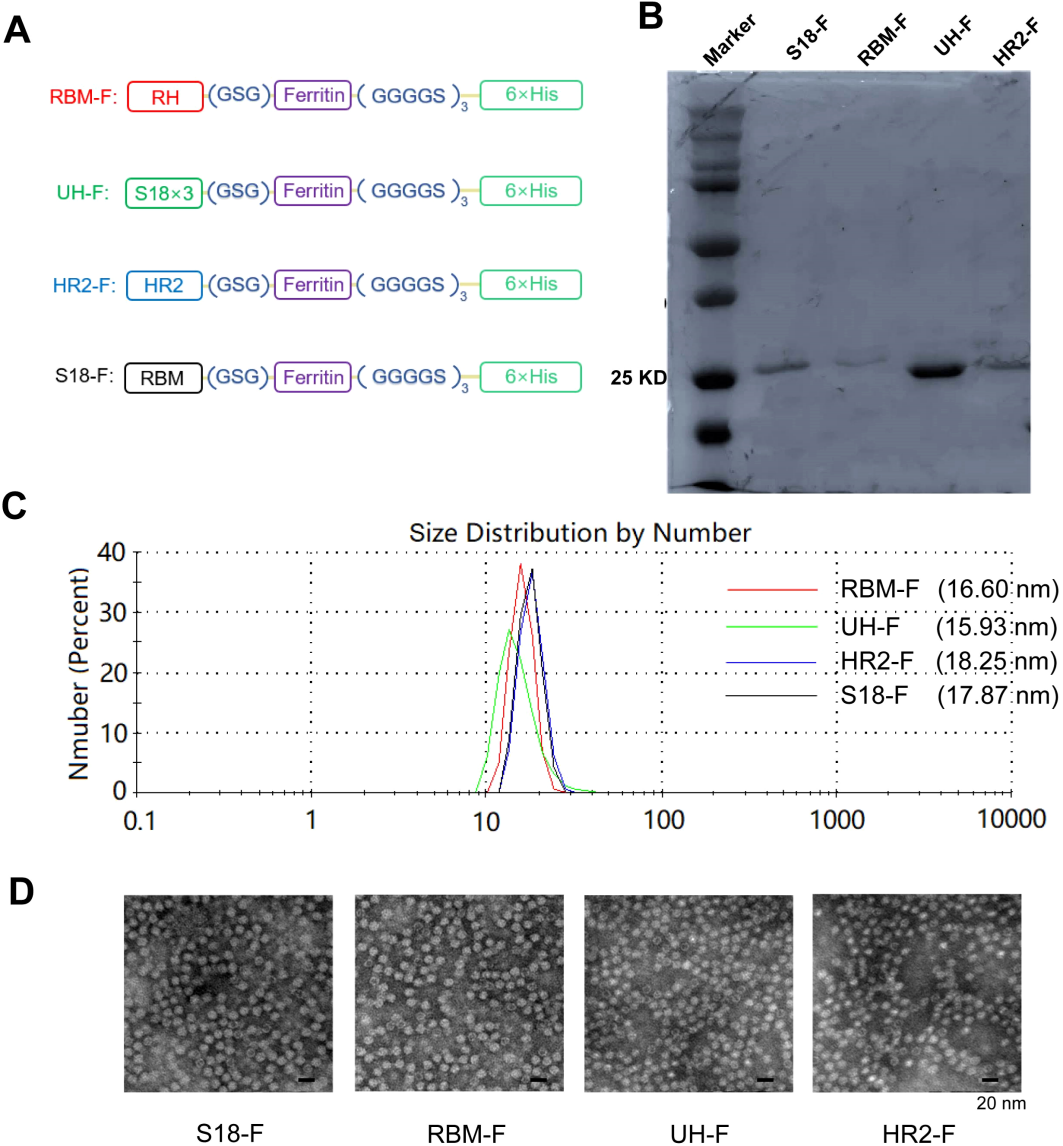
Characterization of self-assembled epitope-based NPs. **(A)** Schematic of conserved epitope-based NPs. **(B)** SDS-PAGE analysis of NPs. **(C)** DLS size distribution of NPs. **(D)** TEM images of NPs.

RBM (71 aa), UH (40 aa), HR2 (42 aa), or a triple-copy of S18 (78 aa) were individually fused to the N-terminus of ferritin in an antigen-ferritin tandem format using a GSG linker. Subsequently, a 6× His-tag was attached via a flexible (GGGGS)_3_ linker at the C-terminus of ferritin (**Figure 2A**).

### 3.2 Self-assembled epitope-based NPs detected *in vitro*

Protein molecular weight and purity were identified by SDS-PAGE and Coomassieblue staining. The theoretical molecular weights of these NP monomers are as follows: S18-F (27.44 kDa), RBM-F (26.77 kDa), UH-F (23.41 kDa), and HR2-F (23.37 kDa). The molecular weights of each NP monomer were approximately consistent with the theoretical values. The purity of the NPs exceeded 90% (**Figure 2B**).

Particle size detection results (**Figure 2C**) indicated that the diameters of NPs were approximately 17.87 nm, 16.6 nm, 15.19 nm, and 18.25 nm, respectively. The size and morphology of the NPs were further characterized using TEM.

The NPs exhibited a hollow, spherical structure resembling ferritin protein (**Figure 2D**). The particle diameter ranged approximately from 10 to 20 nm, consistent with the particle size detection results. These findings indicate successful expression of ferritin-supported NPs in the *E. coli* system, where they self-assembled into NPs. Importantly, the addition of epitope did not impair ferritin’s self-assembly capability.

### 3.3 Strong humoral immune responses-induced by NP antigen

**Figure 3:**
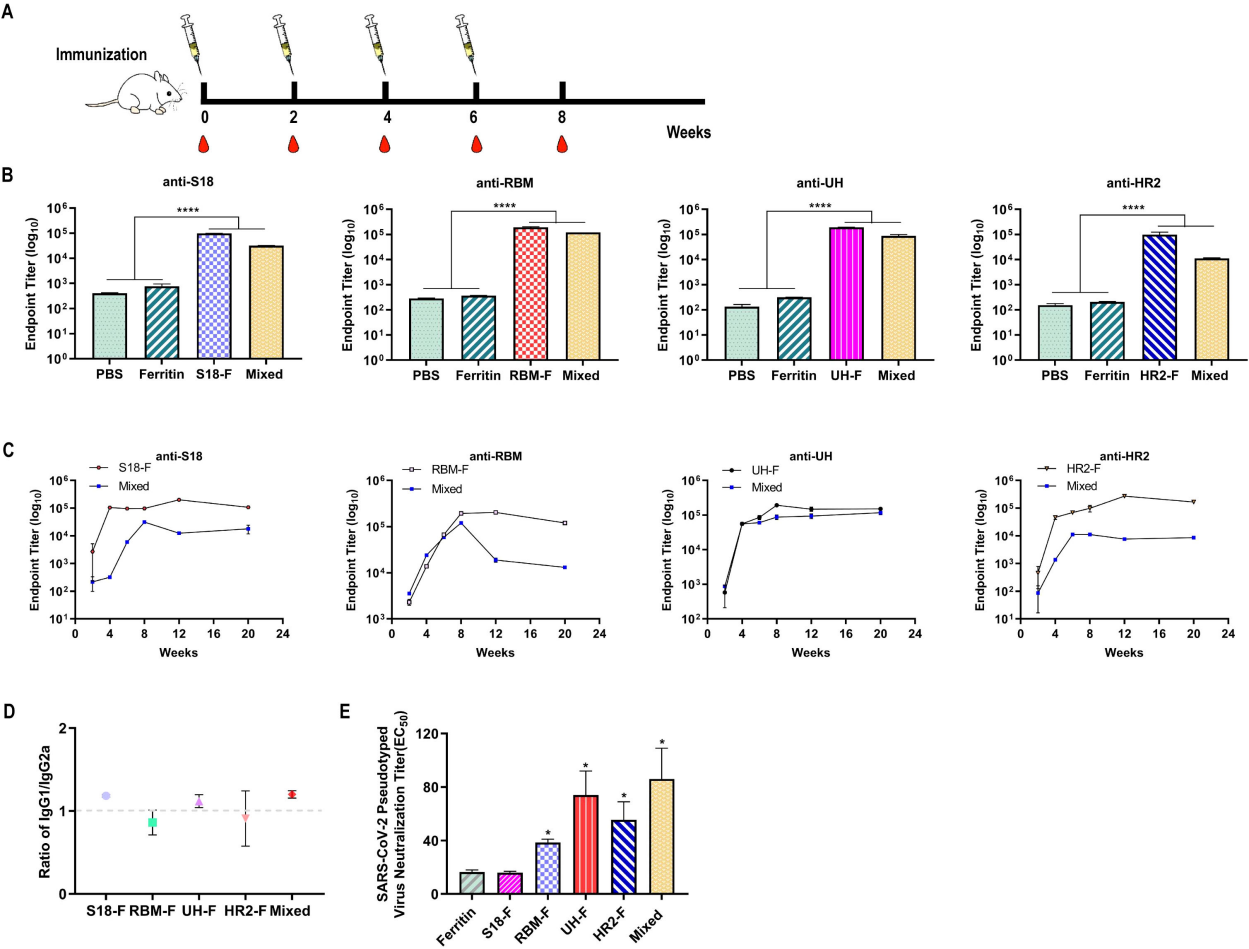
Epitope-based NPs induced robust humoral immune responses in mice. **(A)** Schematic diagram of immunization, and blood collection in BALB/c mice. **(B)** Epitope-specific IgG titers in sera collected two weeks after the final immunization (n = 6). **(C)** Epitope-specific IgG titers in sera measured from week 0 to week 20 following the initial immunization (n = 6). **(D)** The ratio of epitope-specific antibodies in sera. **(E)** Serum neutralizing antibody titers against SARS-CoV-2 pseudovirus WH-1 (n = 6). (ns, not significant; *P < 0.05, ****P < 0.0001, compared to the ferritin group).

To assess the immunogenicity of NPs, all mice underwent four vaccinations at 2-week intervals in a prime-boost regimen (**Figure 3A**). Serum samples were collected every two weeks, and titers of epitope-specific IgG were evaluated using ELISA. The NPs elicited robust titers of epitope-specific antibodies, especially in the RBM-F and UH-F groups. By 8 weeks, specific IgG titers exceeded 10^5^, significantly higher than those in the PBS- and ferritin-immunized groups (P < 0.0001) (**Figure 3B**). Strong epitope-specific immune responses were also observed in the mixed-immunized group. However, due to the lower immunization dose of each NP (5 μg per NP), the titers of specific antibodies for each epitope were relatively diminished compared to the group immunized with NPs alone. This underscores the critical need for future research to refine multi-epitope NP formulations with the aim of enhancing overall immunogenicity.

To assess the long-term changes in antibody levels, serum samples were collected at 12 and 20 weeks post-primary immunization to track the dynamics of antigen-specific antibodies. As shown in **Figure 3C**, the epitope-specific antibody levels in the S18-F and HR2-F groups peaked at 12 weeks and then slightly decreased but remained high. In contrast, the epitope-specific antibody levels in the RBM-F and UH-F groups peaked two weeks after immunization, with the RBM-F group showing a continued decrease by week 12. For the mixed-immunized group, antibody levels against all epitopes peaked two weeks after the four-week immunization period and then began to decline. In summary, all NP immunogens induced specific antibodies that maintained high binding titers for three months following the last immunization.

The antibody titers of epitope-specific IgG1 and IgG2a were evaluated using an antibody isotyping assay to assess the Th1/Th2 bias in the immune response. The IgG1/IgG2a ratio was calculated to indicate the relative balance between Th1 and Th2 immune responses (Lee et al., 2018; Kim et al., 2020). As shown in **Figure 3D**, the IgG1/IgG2a values in the immunized mouse sera in the S18-F, RBM-F, UH-F, HR2-F, and mixed-immunized groups were close to 0.99, indicating that NPs immunization induced relatively balanced Th1 and Th2 immune responses.

Neutralizing antibodies bind to the surface antigen of a virus, preventing it from adhering to target cell receptors and thus blocking infection. These antibodies are crucial indicators of vaccine-induced humoral immunity. In this study, we employed a pseudovirus neutralization test to determine the neutralization titer of immunized sera. The results indicated that compared to the ferritin-immunized group, serum from the S18-F group did not exhibit a neutralizing effect against the SARS-CoV-2 pseudovirus WH-1 (**Figure 3E**). However, the neutralization titers in the RBM-F, UH-F, HR2-F, and mixed-immunized groups were significantly higher than those in the ferritin-immunized group (*P* < 0.05), with the mixed-immunized group demonstrating the highest neutralization capacity.

### 3.4 Potent cellular immune responses induced by NPs in mice

**Figure 4:**
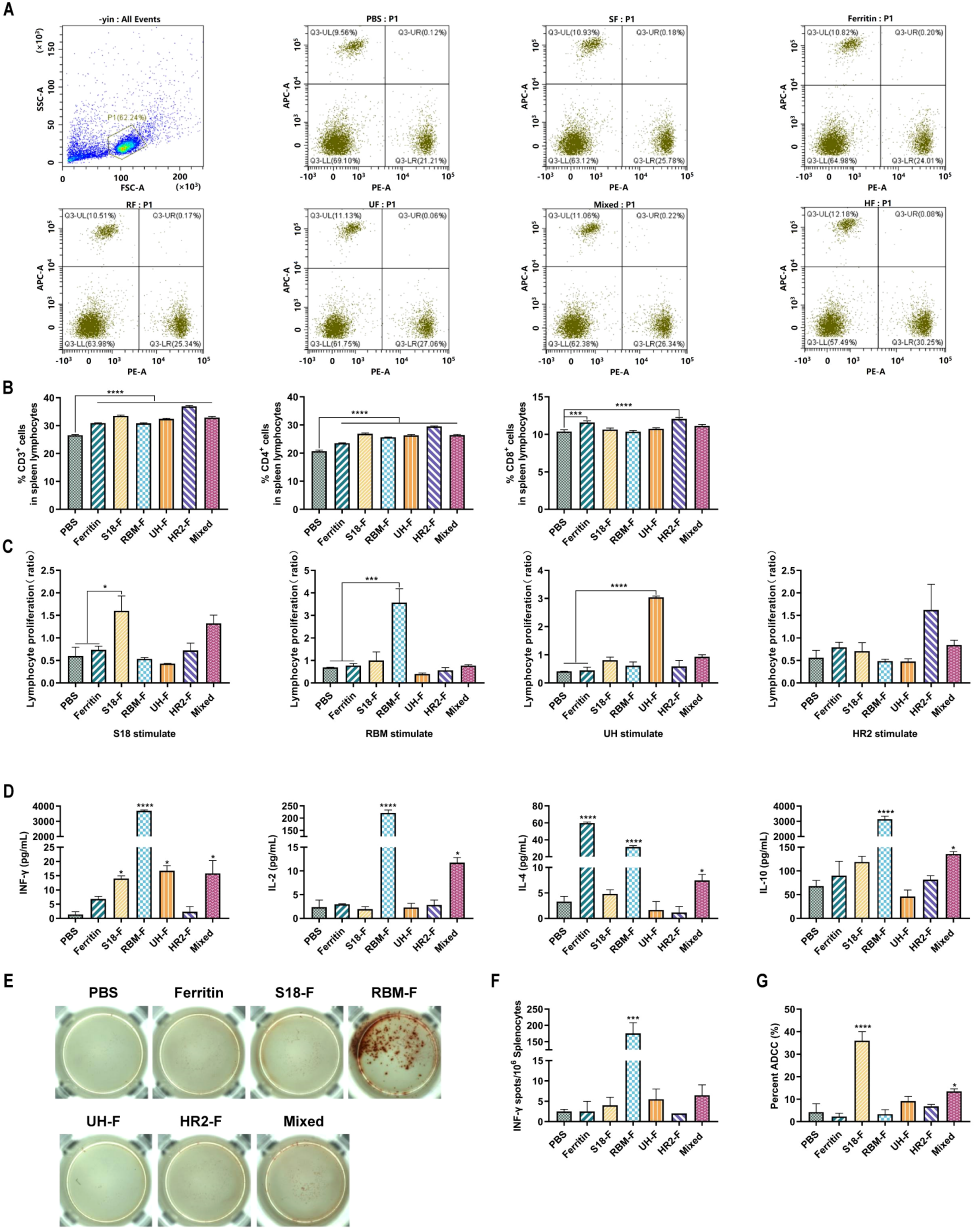
Epitope-based NPs induced robust cellar immune responses in mice. **(A)** Representative flow cytometric analysis of CD3^+^CD4^+^ and CD3^+^CD8^+^ T cells in the splenic lymphocytes. **(B)** Quantification of CD3^+^, CD4^+^, and CD8^+^T cell proportion in the splenic lymphocytes (n = 6). **(C)** Peptide-stimulated splenic lymphocyte proliferation levels. **(D)** The concentrations of IL-2, IL-4, IL-10 and IFN-γ in serum (n= 6). **(E)** The spot wells of IFN-γ cytokine-secreting lymphocytes. **(F)** IFN-γ-secreting lymphocytes isolated from spleens were determined by ELISpot (n = 6). **(G)** ADCC effect of serum (n = 6). (ns, not significant; *P < 0.05, ***P < 0.001, ****P < 0.0001).

CD3+, CD4^+^, and CD8^+^ T cells in the splenic lymphocytes of mice were stained two weeks after the fourth immunization, and the number of these T cells in the splenic lymphocytes was detected by flow cytometry. As shown in **Figures 4A**, **B**, CD3^+^ and CD4^+^ T cells were significantly increased in all NP-immunized groups compared to the PBS-immunized group. A significant increase in the number of CD8^+^ cells was observed in the HR2-F group. These results indicate that specific epitopes stimulate T cell activation and proliferation.

Lymphocytes are important cellular components of the immune response in the body. A series of changes in cell proliferation occurs when stimulated by peptides *in vitro*. The lymphocyte proliferation rate can determine the reactivity and functional status of lymphocytes to relevant stimuli, and is one of the indicators used to evaluate cellular immunity. Two weeks after the last immunization, splenic lymphocyte proliferation in immunized mice was stimulated by polypeptides, and proliferation was detected using the MTT assay. As shown in **Figure 4C**, the splenic lymphocytes of mice in the S18-F, RBM-F, and UH-F groups were significantly increased only under the stimulation of specific epitope peptides compared with those in the PBS-immunized group, whereas no clear proliferation was observed under the stimulation by other peptides. Splenic lymphocytes in the HR2-F group also proliferated slightly upon stimulation by the HR2 peptide. These results indicated that specific epitopes can activate lymphocytes in mice and induce cellular immune responses.

Cytokine secretion by splenic lymphocytes after epitope stimulation was detected using a Th1/Th2 cytokine detection kit. As shown in **Figure 4D**, IFN-γ secretion increased in S18-F and UH-F groups, which may induce TH1-biased immune response. IFN-γ, IL-2, IL-4 and IL-10 cytokines secreted by splenic lymphocytes of mice in RBM-F and mixed-immunized groups were significantly increased, indicating that RBM-F and mixed protein immunization may induce balanced Th1 and Th2 immune responses, which was consistent with the results of antibody typing analysis.

The number of splenic lymphocytes secreting IFN-γ was detected using the ELISPOT method. As shown in **Figures 4E**, **F**, only splenic lymphocytes from RBM-F immunized mice exhibited a significant increase in IFN-γ secretion upon stimulation with the RBM epitope compared to the control group (P < 0.001). The number of lymphocytes secreting IFN-γ in S18-F, UH-F and mixed-immunized groups was slightly higher than that in PBS-immunized group, but the difference was not statistically significant.

ADCC is an important immune defense mechanism of the immune system against viral infections. By specifically binding to the surface antigens of target cells, antibodies recruit effector cells such as natural killer (NK) cells to kill target cells. In this study, the immunized serum-mediated effects of ADCC were examined in mice. The results indicate that the ADCC effect in the UH-F, HR2-F, and mixed-immunized groups was slightly higher compared to the PBS-immunized group (**Figure 4G**), but this difference did not reach statistical significance. Among all the immunized sera, the S18-F-immunized group showed the highest ADCC activity.

### 3.5 NP safety in mice

**Figure 5:**
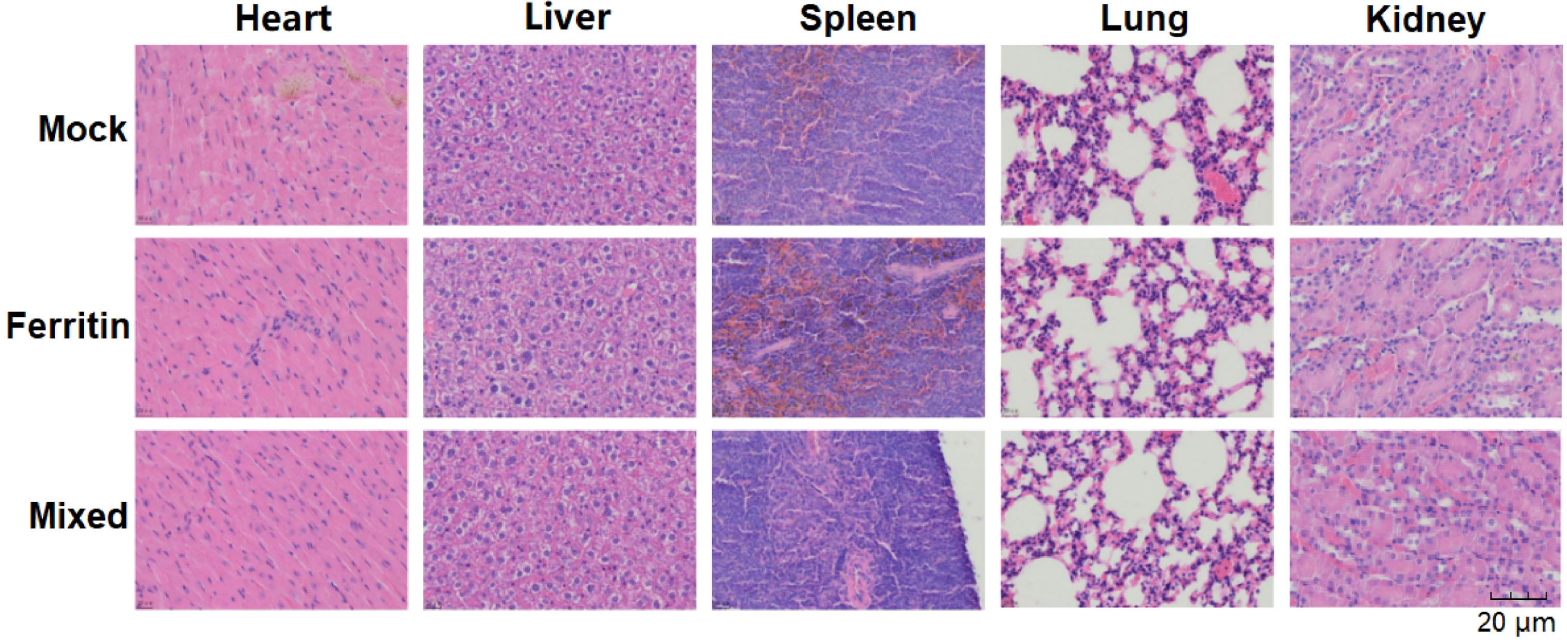
H&E staining of mouse tissue histopathological sections.

Histopathological sections of major organs from mice, including the heart, liver, spleen, lungs, and kidneys, were examined using H&E staining to assess the safety profile of the NPs developed in the experiment. As shown in **Figure 5**, no significant pathological changes were observed in the major organ tissues of mice injected with mixed-immunized NPs, indicating the safety of NPs both when used alone and in combination.

## 4 Discussion

SARS-CoV-2 variants have emerged (Greaney et al., 2021), posing challenges for vaccines targeting S or RBD proteins. Epitope-based vaccines offer advantages, such as rapid production in *E. coli* and the ability to adjust specific epitopes (Li et al., 2014) to counter new strains. They can induce targeted immune responses while minimizing adverse reactions. However, these vaccines face challenges due to epitope instability and lower immunogenicity, necessitating carefully designed adjuvants and delivery systems.

In this study, immunoinformatics tools were used to screen potential epitope sequences (S18, RBM, UH, and HR2). A self-assembling NP vaccine was designed using ferritin as a carrier. Due to variations in immunogenicity among these epitopes, the time required for specific antibodies to reach peak levels varied across groups that might be crucial for optimizing vaccine design and efficacy.

To evaluate the immunogenicity of these epitope NPs, mice were immunized four times at two-week intervals. Mice immunized with NPs produced high-titer specific antibodies against the targeted epitopes, with antibody titers exceeding 10^4^. In contrast, mice in the control group immunized with ferritin or PBS did not produce specific antibodies against these epitopes. Qi S et al. observed significant differences in binding antibody titers in the plasma of New Zealand white rabbits immunized with different epitopes, with some epitopes failing to induce binding antibody production (Sun et al., 2022).

Long-term monitoring of specific antibody levels is influenced by various factors, including the induction of targeted antigens, engagement of immune response mechanisms—especially CD4^+^ and CD8^+^ T cells—and the generation of memory cells (Tillmann et al., 2022; Perez-Saez et al., 2023). Specific antibody titers were sustained for three months in the serum of immunized mice. Although a fourth immunization might sustain antibody production, our findings suggest that the long-term effect is not solely dependent on the fourth immunization. These results indicate that the four epitope-based NPs can induce potent and long-lasting antigen-specific humoral immune responses.

Antibody typing was conducted to determine the types and subtypes of antibodies produced, with the ratio of IgG1 to IgG2a reflecting the immune response bias. Analysis of antibody types in the serum of mice immunized with NPs revealed a close to 1 ratio of IgG1 to IgG2a, indicating a balanced Th1 and Th2 immune response induced by NPs. However, it is important to note that relying solely on IgG1 and IgG2a ratios may not suffice; comprehensive analysis requires integration with other indicators to fully understand the immune response.

Neutralizing antibodies serve as critical indicators of viral vaccine efficacy, capable of binding to SARS-CoV-2 and preventing viral cell infection. This study evaluated the serum’s ability from immunized mice to neutralize SARS-CoV-2 using a pseudovirus neutralization assay. Results showed that mice immunized with PBS, ferritin, and S18-F did not produce neutralizing antibodies against the wild-type pseudovirus WH-1. In contrast, sera from the RBM-F, UH-F, HR2-F, and mixed-immunized groups effectively neutralized pseudovirus WH-1. Despite RBM being considered the primary region for inducing neutralizing antibodies against SARS-CoV-2 (Hastie et al., 2021), the RBM epitope used in this study did not yield high neutralizing antibody titers. Previous studies (Long et al., 2022) have shown that sera from mice immunized with RBM did not exhibit RBD-ACE2 blocking activity, in contrast to sera from monkeys immunized with RBM, which demonstrated neutralizing antibody titers. Comparative studies between different animal models, including primates and rodents, could continue to elucidate species-specific immune responses and further refine vaccine development strategies against emerging viral variants.

Predicting potential binding peptides of mouse major histocompatibility complex (MHC) II molecules for the entire RBD revealed that mouse MHC II molecules had significantly weaker recognition capabilities for the RBM epitope compared to human MHC II molecules. This suggests potential species differences in RBM epitope antigenicity and immunogenicity.

Cellular immunity is crucial for evaluating the efficacy of antiviral vaccines. In this study, splenic lymphocytes from immunized mice were stimulated with specific antigens to assess immune responses. CD3^+^, CD4^+^, and CD8^+^ T cells, fundamental subgroups in immune responses, were analyzed. Post-immunization, all groups showed significant increases in CD3^+^ and CD4^+^ T cells, with the HR2-F group notably increasing CD8^+^ T cells, enhancing viral resistance.

Stimulation with RBM epitopes in the RBM-F group led to increased secretion of IFN-γ, IL-2, IL-4, and IL-10 cytokines by splenic lymphocytes. S18-F and UH-F immunizations enhanced antiviral capabilities primarily through increased IFN-γ levels. Furthermore, ADCC assays demonstrated that sera from S18-F and mixed-immunized groups exhibited ADCC activity, indicating their ability to bind to infected cell surface antigens and activate NK cells or leukocyte Fc-γ receptors to kill infected cells.

Serum from the UH group demonstrated potent neutralization of the SARS-CoV-2 pseudovirus, highlighting its substantial neutralizing capability. Located within the S2 subunit, the UH epitope elicited the highest titers of neutralizing antibodies and exhibits greater conservation across SARS-CoV-2 variants. Targeting this epitope may confer broad cross-neutralizing activity against diverse variants of SARS-CoV-2, though further experimental validation is needed.

The mixed-immunized group demonstrated robust and comprehensive immune responses, characterized by the highest neutralizing antibody titers among all groups, indicating enhanced cross-neutralizing potential. Despite lower specific antibody titers for individual epitopes due to reduced doses (5 μg per epitope), the group exhibited balanced Th1/Th2 immune responses, significant T cell activation (increased CD3^+^ and CD4^+^ T cells), and durable humoral immunity lasting up to three months post-immunization. Additionally, the mixed-immunized group showed potent ADCC activity and broad-spectrum neutralizing capabilities, suggesting that multi-epitope nanoparticles might synergistically induce a more comprehensive and effective immune response against SARS-CoV-2.

Single-epitope immunogens induced limited neutralizing antibody titers, suggesting that enhanced efficacy could be achieved through optimized combinations of multiple epitopes. Xiancai Ma et al. used ferritin to deliver full-length RBD and HR domains of SARS-CoV-2, inducing neutralizing antibodies in pseudovirus assays (Ma et al., 2020). This highlights the potential of RBD and S2 domains to elicit broad-spectrum neutralizing antibodies and cross-reactive immune responses. However, no significant difference in neutralizing antibody titers was found between mixed- and single-protein immunized groups. This emphasizes the potential of conserved linear epitopes derived from the S protein in the development of multi-epitope SARS-CoV-2 vaccines. In our study, the mixed-immunized group, containing multiple effective epitopes, induced higher neutralizing antibody titers against the SARS-CoV-2 pseudovirus (WH-1) compared to single-epitope immunized groups. This suggests that multi-epitope nanoparticles (NPs) may synergistically elicit a more comprehensive and effective immune response against SARS-CoV-2. Future research will optimize these epitopes for a multi-epitope nanoparticle (NP) vaccine and evaluate its cross-protective efficacy through challenge experiments using variants of concern (VOCs) and bat coronaviruses (bat-CoVs). Additionally, evidence suggests that ferritin carriers might enhance mucosal immune responses, potentially serving as self-adjuvants in mucosal immunity (Shah et al., 2021). Verification of these epitope NPs’ effectiveness in eliciting immune responses awaits further study.

## 5 Conclusion

Potential epitopes of the S protein were predicted using immunoinformatics. Recombinant epitopes from the S protein were fused with ferritin NPs and produced using an *E. coli* expression system. Subcutaneous immunization of BALB/c mice with these NPs successfully induced robust epitope-specific humoral immunity. The RBM-F, UH-F, and HR2-F also elicited potent neutralizing antibodies, which persisted for more than 3 months’ post-immunization. The RBM-F, UH-F, and HR2-F demonstrated robust neutralizing antibody responses against the WH-1 pseudovirus. Moreover, stimulation with corresponding epitopes significantly enhanced splenic lymphocyte proliferation in the S18-F, RBM-F, and UH-F groups, underscoring the promotion of vigorous cellular immune responses by these epitopes. The RBM epitope induced increased secretion of IFN-γ, IL-2, IL-4, and IL-10 cytokines in lymphocytes, suggesting its capacity to elicit a balanced Th1/Th2 immune response. Furthermore, immune sera from the S18-F and mixed-immunized groups exhibited substantial ADCC activity, indicating enhanced splenic cell-mediated recognition and elimination of infected cells. As This study demonstrates that ferritin NPs, incorporating selected epitopes of the SARS-CoV-2 S protein, induce strong and durable immune responses in mice. These findings underscore the potential of this approach for developing effective vaccines against COVID-19.

## Data Availability

The raw data supporting the conclusions of this article will be made available by the authors, without undue reservation.
