# Supplementary material for: Self-assembled epitope-based nanoparticles targeting the SARS-CoV-2 spike protein enhanced the immune response and induced potential broad neutralizing activity
Source: Front Cell Infect Microbiol. 2025 Apr 9;15:1560330. doi: 10.3389/fcimb.2025.1560330 (PMC12014594; doi:10.3389/fcimb.2025.1560330)
Supplement: Supplementary file 1 [file Table1.pdf]

Supplementary Material

Supplementary Table

Supplementary Table 1. Location of epitopes in the RBD/S2 domains and their corresponding positions in the full-length S protein.

Epitope	Residues in RBD/S2 domain	Residues in Spike protein
S18	RBD_{84-109}	$S_{416-441}$
RBM	$RBD_{106-176}$	$S_{438-498}$
UH	$S2_{431-470}$	$S_{1133-1172}$
HR2	$S2_{460-501}$	$S_{1162-1203}$
